# Supplementary material for: Identifying predictors and determining mortality rates of septic cardiomyopathy and sepsis-related cardiogenic shock: A retrospective, observational study
Source: PLoS One. 2024 Apr 25;19(4):e0299876. doi: 10.1371/journal.pone.0299876 (PMC11045062; doi:10.1371/journal.pone.0299876)
Supplement: S1 Table — (DOCX) [file pone.0299876.s001.docx]

| Lab | Abnormal Values | |
| --- | --- | --- |
|  | Low | High |
| Hemoglobin | Female < 12 g/dL  Male < 13.5 g/dL | Female > 16 g/dL  Male > 17.5 g/dL |
| Platelet count | < 150 K/µL | > 400 K/µL |
| Troponin I | - | > 0.04 ng/mL |
| White blood cells |  |  |
| Age < 20 | < 4.5 K/uL | > 12.5 K/uL |
| Age = 20 | < 4.5 K/uL | > 11.5 K/uL |
| Age > 20 | < 3.6 K/uL | > 10.6 K/uL |
