# Supplementary material for: Identifying predictors and determining mortality rates of septic cardiomyopathy and sepsis-related cardiogenic shock: A retrospective, observational study
Source: PLoS One. 2024 Apr 25;19(4):e0299876. doi: 10.1371/journal.pone.0299876 (PMC11045062; doi:10.1371/journal.pone.0299876)
Supplement: S2 Table — (DOCX) [file pone.0299876.s002.docx]

| Characteristic | Septic Shock | Septic Cadiomyopathy | SeRCS |
| --- | --- | --- | --- |
|  | n=977 | n=207 | n=45 |
| Left ventricular ejection fraction (%) | 65.0 (55.0-70.0) | 35.0 (26.5-41.5) | 27.0 (18.0-35.0) |
| Cardiac index (L/min/m²) | - | 2.5 (1.9-3.2) | 1.6 (1.2-1.8) |
| Inotrope Use | 187 (19.1%) | 80 (38.6%) | 21 (46.7%) |
| Inotropes administered after echocardiogram | 126 (67.4%) | 65 (81.3%) | 15 (71.4 %) |
| Intensive care unit length of stay | 6.7 (3.6-12.5) | 6.9 (3.0-12.6) | 6.7 (4.4-10.1) |
| Ventilator free days | 28.0 (8.0-28.0) | 28.0 (9.9-28.0) | 28.0 (7.0-28.0) |
| Elixhauser components |  |  |  |
| History of congestive heart failure | 346 (35.4%) | 159 (76.8%) | 42 (93.3%) |
| History of cardiac arrhythmias | 634 (64.9%) | 141 (68.1%) | 39 (86.7%) |
| History of cardiac valvular disease | 248 (25.4%) | 79 (38.2%) | 31 (68.9%) |
| History of pulmonary circulatory  disorders | 310 (31.7%) | 66 (31.9%) | 25 (55.6%) |
| History of peripheral vascular disorders | 240 (24.6%) | 71 (34.3%) | 27 (60.0%) |
| History of hypertension | 762 (78.0%) | 158 (76.3%) | 38 (84.4%) |
| Blood chemistries |  |  |  |
| Peak troponin (ng/ml) | 0.1 (0.0-0.6) | 0.3 (0.1-1.6) | 0.3 (0.1-1.2) |
| Peak brain natriuretic peptide (ng/L) | 294.0 (104.0-825.5) | 1041.0 (385.2-2189.8) | 1289.5 (659.2-2318.0) |
| White blood cells (/uL) | 12.2 (4.7-21.4) | 9.2 (3.2-20.8) | 12.9 (7.9-16.5) |
| Hemoglobin (g/dL) | 11.9 (10.2-13.9) | 12.9 (10.6-14.8) | 12.9 (11.9-14.1) |
| Platelets (/µL) | 184.0 (120.0-265.0) | 183.0 (124.0-262.5) | 183.0 (130.0-241.0) |
