# Supplementary material for: Identifying predictors and determining mortality rates of septic cardiomyopathy and sepsis-related cardiogenic shock: A retrospective, observational study
Source: PLoS One. 2024 Apr 25;19(4):e0299876. doi: 10.1371/journal.pone.0299876 (PMC11045062; doi:10.1371/journal.pone.0299876)
Supplement: S3 Table — (DOCX) [file pone.0299876.s003.docx]

| Gram Stain Result | Septic Shock | Septic Cardiomyopathy | SeRCS |
| --- | --- | --- | --- |
| Gram positive | 174 (17.8%) | 50 (24.2%) | 10 (22.2%) |
| Gram negative | 213 (21.8%) | 45 (21.7%) | 8 (17.8%) |
| Multiple organisms | 65 (6.7%) | 14 (6.8%) | 1 (2.2%) |
