# Supplementary material for: Identifying predictors and determining mortality rates of septic cardiomyopathy and sepsis-related cardiogenic shock: A retrospective, observational study
Source: PLoS One. 2024 Apr 25;19(4):e0299876. doi: 10.1371/journal.pone.0299876 (PMC11045062; doi:10.1371/journal.pone.0299876)
Supplement: S4 Table — (DOCX) [file pone.0299876.s004.docx]

| Feature List | Total # of Feature Lists | Frequency |
| --- | --- | --- |
| SCM | 2700 |  |
| Body mass index, history of congestive heart failure |  | 84 |
| SeRCS | 1200 |  |
| History of peripheral vascular disorders, history of valvular disease, history of congestive heart failure, history of coronary artery disease, history of pacemaker |  | 51 |
