# Supplementary material for: Identifying predictors and determining mortality rates of septic cardiomyopathy and sepsis-related cardiogenic shock: A retrospective, observational study
Source: PLoS One. 2024 Apr 25;19(4):e0299876. doi: 10.1371/journal.pone.0299876 (PMC11045062; doi:10.1371/journal.pone.0299876)
Supplement: Attachment — Submitted filename: Revision Response.docx [file pone.0299876.s005.docx]

**Editor’s Comments**

Reviewer #1

1. Inclusion of data of over 10 years duration has added a lot of granularity in the data.

This is an important point. To enroll a sufficient number of patients to make meaningful conclusions required a long look-back period. Fortunately, IHC has a decades-long history of maintaining a robust electronic data warehouse which greatly reduces the granularity one would expect to find in other health systems.

2. Is it possible to look into Takotsubo phenotype among the SCM group. It is quite understandable that it may not be logistically feasible given the retrospective nature of the study design.

This comment has been taken to heart. Unfortunately, we would not be able to accurately review all records to make the diagnosis of Takotsubo phenotype given limitations in data available in this retrospective study. We have added to the last paragraph of the discussion section:

“Given the limitations of the retrospective nature of this study Takotsubo phenotype was not specifically identified and it is possible the condition represents a portion of the SCM and SeRCS cohorts.”

3. Since the 2000s there has been evolution in critical care practices. The rise and fall of the fluid resuscitation in sepsis and septic shock. There has been growing attention to minimization of sedation, early mobilization, and sepsis recognition and treatment, the latter of which may mitigate mortality due to sepsis. Because of these changes in practices it could have potentially impacted the clinical outcomes of interest. It would be important to acknowledge this in the manuscript specially given the retrospective nature of the study design.

To address this important point, we have added to the final paragraph of the discussion section:

“Over the course of the study duration, much has changed in the diagnosis and treatment of sepsis and septic shock in critical care which increases the possibility of non-random biases influencing key parameters and outcomes of this study.”

Reviewer #2

1. Clearly define the time frame within which a previous echocardiogram must have been conducted to be considered valid for comparison. Considering the natural progression of cardiac function over time, a consensus on what constitutes an appropriate 'baseline' echocardiogram should be established.

The reviewer rightly notes an ongoing problem with all cohorts defined by ICU admission. The methods employed in our study are an improvement upon other work in the field, but the reviewer is correct that limitations remain. We have added the following text to the manuscript to acknowledge and engage this reviewer’s concern. We have included this limitation in the final paragraph of the discussion section:

“A key limitation of this and prior research among critically ill patients is the lack of perfect knowledge of the patient’s comorbid state immediately prior to onset of sepsis. Some patients who appear to have septic cardiomyopathy may in fact have another etiology of their cardiac dysfunction. As an improvement upon other studies in this field, we did clinically review pre-admission echocardiograms. The security of our observed findings is thus higher than in many other studies in this field, but large, prospective, community-based cohorts would be required to answer this question definitively.”

2. In the absence of a recent echocardiogram, additional diagnostic criteria should be employed to differentiate SCM from preexisting cardiac conditions. This could include markers of cardiac strain related to sepsis, biomarkers, or a comprehensive review of the patient’s medical history of documented cardiac issues.

This point is well taken. We analyzed cardiac and biomarkers as well as reviewed charts to exclude patients who had presented with a primary cardiac abnormality rather than septic shock. All patients in the study had septic shock as their primary pathology and given the limitations of a retrospective study, the changes in LVEF were assigned to the septic shock. Please see our comment above for the additional text added to the manuscript.

3. Conduct sensitivity analyses to determine how including patients without recent echocardiograms might affect the study's findings and conclusions. This could help to understand the potential bias introduced by including these patients.

As we have mentioned, obtaining baseline echocardiograms is a limitation of this study. However, the aim of this study was to create a prediction tool that would assist clinicians in identifying patients at risk for SCM and SeRCS. This objective necessitates the use retrospective data that would closely resemble the data a clinician would have access to during an encounter. In many cases, prior echocardiograms are not available to the clinician, nor are there existing tools that would help them to quickly compare a current echocardiogram to a prior one. Without having a reliable method to tease out these patients, we are not able to run a sensitivity analysis. Additionally, this patient cohort is not large and adding an additional subgroup to this cohort would likely not result in significant findings.
